# A survey of the research practice in general medicine departments of Japanese universities: A cross‐sectional study

**DOI:** 10.1002/jgf2.473

**Published:** 2021-06-24

**Authors:** Masaki Tago, Takashi Watari, Kiyoshi Shikino, Shun Yamashita, Naoko E. Katsuki, Motoshi Fujiwara, Shu‐ichi Yamashita

**Affiliations:** ^1^ Department of General Medicine Saga University Hospital Saga Japan; ^2^ General Medicine Center Shimane University Hospital Shimane Japan; ^3^ Department of General Medicine Chiba University Hospital Chiba Japan

**Keywords:** English‐language research publication, general medicine, Japanese university hospital, research practice

## Abstract

**Background:**

Few studies have focused on research practice in Japanese university general medicine (GM) departments.

**Methods:**

This is a questionnaire‐based cross‐sectional study to clarify the research achievement and associated factors of Japanese university GM department. Univariate analysis was performed to compare the number of English‐language research publications and explanatory variables.

**Results:**

Forty‐seven universities responded. Over a 3 years period, the median number of English‐language research publications was 6. Perceived degree of research necessity, staff numbers, collaborative research, conference presentations, and obtaining research grants were significantly associated with a higher number of English‐language research publications.

**Conclusions:**

While GM research output was found to be limited, numerous associated factors can potentially change Japanese GM departments' research environments.

## Graphical abstract

This questionnaire‐based cross‐sectional study showed the median number of English‐language research publications was 6. Research achievements were associated with the perceived degree of research necessity, staff and postgraduate student numbers, collaborations with other facilities, presentations in academic conferences, and obtaining research grants. While GM research output was found to be limited, numerous factors can potentially change Japanese GM departments' research environments.

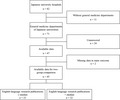

## 1 INTRODUCTION

In Japan, the specialty of general medicine (GM) was started in April 2018 as the 19th basic area in the medical specialty board system. Japanese Medical Specialty Board shows that GM includes hospital medicine, family medicine, and general internal medicine.^1^ Studies have found that research productivity in GM is limited globally as well as Japan.^2^, ^3^, ^4^, ^5^ University departments of GM are expected to contribute to research in addition to clinical practice and medical education to enhance the role and value of GM department.^3^ Previous reports have been published on the practice and role of GM departments in Japanese universities.^6^, ^7^ However, few studies have focused on the research themes in these departments, their contributions to the academic field, and factors associated with wider academic contributions.^8^, ^9^

This study aimed to survey the status of research in the GM departments of Japanese universities and explore factors associated with their research achievements, thereby identifying enablers to enhance research practice.

## 2 METHODS

This was a cross‐sectional questionnaire‐based study. Questionnaires were sent on June 1, 2020, to all 82 universities on the public mailing list of the Council of Japanese University Hospitals for General Medicine. The council covers GM departments in university hospitals throughout Japan, and its annual meeting aims to promote communication and information sharing among Japanese university hospitals. Responses were collected using Google Forms. The department chairperson was responsible for completing the questionnaire, which required department name and chairperson position to be stated. All authors collaboratively developed the questionnaire; it comprised five‐point Likert‐scale, yes–no, descriptive, and numeric questions. The questions and definitions appear in the Appendix S1.

We set the primary outcome of this study as the number of English‐language research publications (ELRP) in the 3 years from 2017 to 2019 because it is an internationally assessable research level. The Spearman correlation coefficient (*r*) was calculated to test correlations with a range of factors. Completed questionnaires were divided into two groups based on the median number of ELRP, and missing values were excluded from the comparative analysis. Continuous variables were expressed using median values and interquartile range and were compared using the Mann–Whitney U‐test. Categorical variables were expressed as percentages and compared using chi‐square tests or Fisher's exact test. Statistical significance was set at *p* < 0.05. IBM SPSS version 25 (IBM Corp., Armonk, NY) was used for statistical analyses.

All subjects gave informed consent on the questionnaire Web site. The Ethics Committee of Saga University Hospital waivered this study because it was not conducted on humans, and did not include personal information, and university names were anonymized in the analysis.

## 3 RESULTS

**FIGURE 1:**
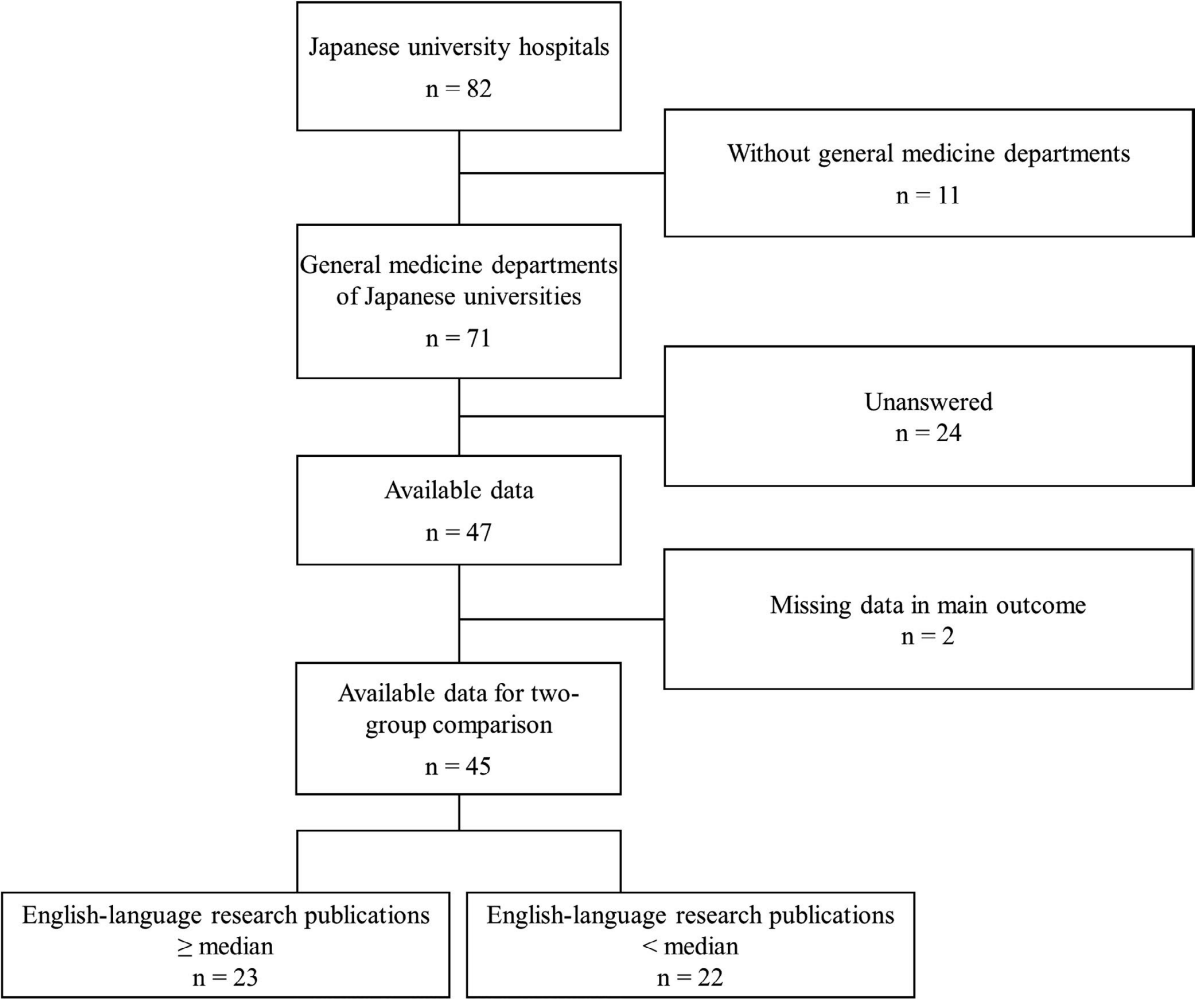
Data flow diagram

In total, 47 universities responded, with a 66.2% response rate (Figure 1). The responses, Spearman correlation coefficient between the number of English‐language research publications and other factors, and results of univariate analysis are shown in Table 1. Over the 3 years from 2017 to 2019 inclusive, the median and total numbers of ELRP, international academic conference presentations, and public research grants received were 6 and 660, 4 and 336, and 2 and 161, respectively. The median number of staff at assistant professor level or above and full‐time physician staff was 6 and 8, respectively.

**TABLE 1:** The responses, the Spearman correlation coefficient, and the results of univariate analysis.

	Median (IQR) *n* = 47	Total sum (*n*)	*r*	English‐language research publications ≥6 *n* = 23 Median (IQR) or *n* (%)	English‐language research publications <6 *n* = 22 Median (IQR) or *n* (%)	*p*
Perceived degree of research necessity in GM (5 levels)	5 (4–5)	NA	0.304*	5 (5–5)	5 (4–5)	0.021
Current status of research in GM (5 levels)	2 (2–3)	NA	NS	3 (2–3)	2 (2–3)	0.943
Actual research effort^a^	15 (10–20)	NA	NS	20 (10–20)	10 (10–20)	0.177
≥30%^a^	NA	7 (16%)	NA	4 (17%)	3 (13%)	1.000
Ideal research effort^a^	30 (30–30)	NA	NS	30 (30–30)	30 (29–33)	0.513
≥30%^a^	NA	38 (84%)	NA	21 (91%)	17 (77%)	0.234
Professor including tenured and nontenured	NA	35 (75%)	NS	16 (70%)	19 (86%)	0.284
Full‐time physician staff^a^	8 (4–14)	526	0.391**	10 (7–19)	6 (4–11)	0.011
Assistant professor or above^a^	6 (3–8)	341	0.400**	7 (5–12)	4 (2–6)	0.003
Female assistant professors or above^b^	1 (0–3)	84	0.342*	2 (1–4)	1 (0–2)	0.016
Medical staff and residents^a^	3 (0–7)	185	NS	3 (1–7)	2 (0–5)	0.130
Postgraduate students^a^	1 (0–3)	105	0.483**	2 (0–4)	0 (0–2)	0.027
Full‐time researchers^b^	0 (0–0)	13	0.437**	0 (0–1)	0 (0–0)	0.049
Research education system^a^	NA	20 (44%)	NS	13 (59%)	7 (32%)	0.069
Research conducted at other sites^b^	NA	20 (44%)	0.459**	14 (64%)	6 (27%)	0.015
Collaborative research^a^	NA	23 (51%)	0.438**	16 (70%)	7 (32%)	0.011
International academic conference presentations (3 years)^a^	4 (0–9)	336	0.569**	6 (2–15)	1 (0–5)	0.002
Domestic and international academic conference research presentations (3 years)^a^	8 (5–24)	929	0.676***	22 (7–40)	5 (4–9)	0.001
English‐language research publications (3 years)^a^	6 (3–23)	660	NA	NA	NA	NA
Japanese‐language research publications (3 years)^a^	1 (1–2)	137	0.350*	2 (0–3)	1 (0–1)	0.057
Public research grants received (3 years)^b^	2 (1–3)	161	0.722***	3 (2–12)	1 (0–2)	<0.001
Commercial research grants received (3 years)^c^	4 (1–18)	462	0.446**	9 (3–22)	2 (0–5)	0.019

Abbreviations: GM, general medicine department in Japanese universities; IQR, interquartile range; NA, not applicable; NS, not significant; r, Spearman correlation coefficient.

^a^ *n* = 45.

^b^ *n* = 44.

^c^ *n* = 41.

All available data were used, and missing data were removed.

* *p* < 0.05.

** *p* < 0.01.

*** *p* < 0.001.

Correlations were found between numbers of English‐language research publications and public research grants received (|*r*| > 0.7), and other factors, including number of staff at assistant professor level or above, postgraduate students, full‐time researchers, collaborative research, international conference presentations, research presentations, and commercial research grants (0.4 < |*r*| ≤ 0.7; Table 1).

In the univariate analysis, the group with the higher number of ELRP had a significantly higher number or percentages of the following: the perceived degree of research necessity in GM; numbers of full‐time physicians; staff at assistant professor or above; female staff at assistant professor or above; postgraduate students; full‐time researchers; amount of research conducted at other sites; amount of collaborative research; and numbers of international academic conference presentations, domestic, and international academic conference research presentations, and public or commercial research grants received.

## 4 DISCUSSION

In this study, the perceived degree of research necessity; number of staff, graduate students, and full‐time researchers; amount of research conducted at other sites; collaborative research; and number of international academic conference presentations, conference research presentations, and public or commercial research grants received were significantly correlated with the number of ELRP. Studies have shown that increasing academic productivity, promoting an academic GM department, and hospitalists' academic success were associated with the following: leaders' vision for the nature of the research enterprise; having graduate degree programs; spending more time on research; collaboration with non‐generalist faculties and non‐physician investigators in research; presenting project results; grant applications; and mentorship.^3^, ^10^, ^11^, ^12^ Those findings and our study results are similar; however, we found that research efforts and the research education system were not significantly associated with the number of ELRP. That may be because research efforts could be judged based on human resources, length of time, and the leaders' personal subjectivity; further, the education system and mentorship do not exactly coincide. These findings illustrate the importance of expertise in conducting research and obtaining research grants and sufficient academic staff numbers for success in ELRP. Because it is difficult to rapidly increase staff numbers, a priority is to develop skills and knowledge associated with conducting research and obtaining research grants among existing staff.^13^

This study showed that only on average (median) six ELRP were published by GM university departments over this 3 year period. A previous report showed a lower rate of ELRP in major international journals associated with primary care^4^, ^5^; therefore, the growth of academic practice in GM in Japan has been strongly expected. The perceived degree of research necessity includes the vision of the nature of the research enterprise, which means conveying the importance of critical inquiry and consistently insisting on a balance of effort of academic activities.^11^ While the considerable necessity for research in GM departments of Japanese universities was significantly correlated with the number of ELRP, no significant relationship was found between research effort and ELRP. These findings suggest that developing the vision for the nature of the research enterprise in Japanese general physicians could lead to an increase in the number of ELRP.

This study has several limitations. It was a cross‐sectional questionnaire‐based study in which some universities did not respond. This study was conducted only in the GM departments of universities' main hospitals; thus, it does not reflect the situation in GM departments at affiliated university hospitals. This limited sample meant that responses may not reflect the situation in all such departments, and the univariate analysis could not account for potential confounding factors. This study identified associated factors and found that improvements in such factors may not necessarily lead to increased ELRP.

## 5 CONCLUSIONS

Research practice in GM departments of Japanese universities was found to be limited. Research achievements were associated with the perceived degree of research necessity, staff and postgraduate student numbers, collaborations with other facilities, presentations in academic conferences, and obtaining research grants. Focusing on these factors could help to establish systems for research education, research collaboration, and research sharing within and between universities and more broadly in the field of GM.

## CONFLICT OF INTEREST

The authors have stated explicitly that there are no conflicts of interest in connection with this article.

## Supporting information

App S1

## References

[1] Japanese Medical Specialty Board, Specialist of General Medicine, Website . https://jmsb.or.jp/sogo#an02. Accessed April 22, 2021. (in Japanese).

[2] Chopra V , Burden M , Jones CD , Mueller S , Gupta V , Ahuja N , et al. State of research in adult hospital medicine: results of a national survey. J Hosp Med. 2019;14(4):207–11. 30933670 10.12788/jhm.3136

[3] Reid MB , Misky GJ , Harrison RA , Sharpe B , Auerbach A , Glasheen JJ . Mentorship, productivity, and promotion among academic hospitalists. J Gen Intern Med. 2012;27(1):23–7. 21953327 10.1007/s11606-011-1892-5 PMC3250536

[4] Aoki T , Fukuhara S . Japanese representation in high‐impact international primary care journals. Off J Jpn Primary Care Assoc. 2017;40:126–30. (in Japanese).

[5] Fukui T , Takahashi O , Rahman M . Japanese representation in leading general medicine and basic science journals: a comparison of two decades. Tohoku J Exp Med. 2013;231(3):187–91. 24189990 10.1620/tjem.231.187

[6] Kita K , Shimizu Y , Yamashiro S . Referral and consultation practice between generalists and specialists at a university hospital: a retrospective cross‐sectional study. Off J Jpn Primary Care Assoc. 2019;42(2):92–7. (in Japanese).

[7] Takeoka H , Horibata K , Masui S , Ajisaka K , Nabeshima S . Trends in department of General Medicine in University Hospitals in Japan Searched from Websites. Med Bull Fukuoka Univ. 2017;44(2):81–6.

[8] Komagamine J , Yabuki T . Full‐text publication rate of abstracts presented at the Japan Primary Care Association Annual Meetings (2010–2012): a retrospective observational study. BMJ Open. 2018;8:e021585. 10.1136/bmjopen-2018-021585 PMC6020981 29934391

[9] Kenzaka T , Kamada M . Barriers to preparation of case reports among Japanese general practitioners. Saudi J Med Med Sci. 2020;8:239–40. 32952518 10.4103/sjmms.sjmms_604_19 PMC7485660

[10] Saha S , Saint S , Christakis DA , Simon SR , Fihn SD . A survival guide for generalist physicians in academic fellowships part 2: preparing for the transition to junior faculty. J Gen Intern Med. 1999;14(12):750–5. 10632820 10.1046/j.1525-1497.1999.12148.x PMC1496859

[11] Friedman RH , Alpert JJ , Green LA . Strengthening academic generalist departments and divisions. J Gen Intern Med. 1994;9(4 Suppl 1):S90–98. 8014750 10.1007/BF02598123

[12] Haas DM . Ten tips for an academic generalist. Obstet Gynecol. 2007;109(5):1184–6. 17470603 10.1097/01.AOG.0000262053.04349.6c

[13] Shannon EM , Chopra V , Greysen SR , Herzig SJ , Kripalini S , O'Leary KJ , et al. Dearth of hospital medicine clinician investigators across United States Medical Centers: a call to action. J Hosp Med. 2021;16(3):189–91. 33617444 10.12788/jhm.3536 PMC7929609

