# Supplementary material for: A survey of the research practice in general medicine departments of Japanese universities: A cross‐sectional study
Source: J Gen Fam Med. 2021 Jun 24;23(1):56–60. doi: 10.1002/jgf2.473 (PMC8721328; doi:10.1002/jgf2.473)
Supplement: Supplementary file 1 — App S1 [file JGF2-23-56-s001.docx]

**Supplement: List of questionnaire items and definitions**

***Five-point Likert-scale questions***

- **Perceived degree of research necessity in GM:** *“In general, research is necessary in university GM department”* (1: strongly disagree, 5: strongly agree)
- **Current status of research in GM:** *“Currently, research is practically conducted in university GM department”* (1: strongly disagree, 5: strongly agree)

***Yes-no questions***

- **Research education system:** Research team, multi-layered education, and mentoring system
- **Research conducted at other sites:** Research at affiliated hospitals and leading multicenter research
- **Collaborative research:** Research collaboration with other universities, public institutions, and research organizations

***Descriptive questions***

- **Actual research effort:** Assuming overall departmental work to be 100%.
- **Ideal research effort:** Assuming overall departmental work to be 100%.

***Numeric questions***

- **Professor:** Tenured and non-tenured professors
- **Full-time physician staff:** All full-time physicians of any position or staff of equivalent position (non-tenured faculty, endowed courses)
- **Assistant professor or above:** Professors, associate professors, lecturers, assistant professors, or staff with equivalent positions (non-tenured faculty, endowed courses)
- **Female assistant professors or above:** Female professors, associate professors, lecturers, assistant professors, or staff with equivalent positions (non-tenured faculty, endowed courses)
- **Medical staff and residents:** Physicians who work full-time in the department, regardless of their employment situation—except for those who are assistant professor or above
- **Postgraduate students:** Postgraduate students in the department
- **Full-time researchers:** Physician or researcher in the department whose research amounts to at least 70% of their work
- **International academic conference presentations (3 years):** Number of presentations at international academic conferences—regardless of conference or presentation type
- **Domestic and international academic conference research presentations (3 years):** Number of research presentations (excluding case reports and activity reports) at domestic and international academic conferences of any type
- **English-language research publications (3 years):** Number of research publications in English excluding reviews and case reports—regardless of journal type
- **Japanese-language research publications (3 years):** Number of research papers in Japanese excluding reviews and case reports—regardless of journal type
- **Public research grants received (3 years):** Number of public research grants awarded, e.g., KAKENHI, health and labor grants-in-aid for scientific research, and AMED
- **Commercial research grants received (3 years):** Number of public research grants awarded by private organizations and companies other than public organizations
